# Myocardial Protection in Isolated Coronary Artery Bypass Grafting: Del Nido *versus* Blood Cardioplegia

**DOI:** 10.21470/1678-9741-2022-0093

**Published:** 2023

**Authors:** Taha Özkara, Mehmet Ali Kayğın, Servet Ergün, Hüsnü Kamil Limandal, Mevriye Serpil Diler, Hatice Işıl Çüçen Dayı, Ziya Yıldız, Özgür Dağ

**Affiliations:** 1 Department of Cardiovascular Surgery, Erzurum Regional Education and Research Hospital, Erzurum, Turkey; 2 Department of Pediatric Cardiovascular Surgery, Erzurum Regional Education and Research Hospital, Erzurum, Turkey

**Keywords:** Cardiac Tamponade, Cardiopulmonary Bypass, Cardiopulmonary Resuscitation, Constriction, Coronary Artery Bypass, Extracorporeal Membrane Oxygenation, Gender Identity, Heart Arrest, Induced.

## Abstract

**Introduction:**

Our study aimed to examine the impacts of blood cardioplegia (BC) and del Nido cardioplegia (DNC) solutions - which we used in isolated coronary artery bypass grafting (CABG) - on early mortality and major adverse events (MAE).

**Methods:**

We retrospectively analyzed 329 consecutive patients who underwent CABG in our clinic between January 2016 and January 2020. Myocardial infarction, reoperation, cardiac tamponade, stroke, renal failure, extracorporeal membrane oxygenation requirement, and cardiopulmonary resuscitation were defined as MAE. The group in which DNC was used was Group D (181 [55%] patients), and the group in which BC was used was Group B (141 [45%] patients).

**Results:**

No statistically significant difference was determined between the groups regarding age, weight, body surface area, gender, or European System for Cardiac Operative Risk Evaluation score (P=0.615, P=0.560, P=0.934, P=0.365, P=0.955, respectively). Although there was no statistically significant difference between the groups in terms of aortic cross-clamping time (P=0.712), cardiopulmonary bypass duration was longer in Group B (P=0.001). Even though the incidence of stroke was higher in Group B (P=0.030), no statistically significant difference was observed between the groups regarding total incidence of MAE, mortality, mechanical ventilation time, length of stay in the intensive care unit, or length of hospital stay (P=0.153, P=0.130, P=0.689, P=0.710, P=0.613, respectively).

**Conclusion:**

We found no significant difference in MAE, mortality, duration of mechanical ventilation, intensive care unit stay, or hospital stay between the DNC and BC groups. We believe that both solutions can be used safely for cardiac protection in the adult patient population.

## INTRODUCTION

Appropriate myocardial protection is the most critical step in open-heart surgery. Although there have been various solutions developed for myocardial protection, the most commonly used solution, especially in the adult patient population, is blood cardioplegia (BC). In recent years, the del Nido cardioplegia (DNC) solution, produced especially for the pediatric age group, has started being used. Successful results have also been reported in the adult patient group over time^[^1^-^7^]^. The DNC solution seems to be a good alternative to BC for myocardial protection of the adult patient population, as it allows longer arrest durations. However, the DNC solution was developed for the pediatric patient population with immature myocardial tissue. Concerns arise as to whether it is sufficient to protect the mature adult myocardium. It is known that the mature myocardium is more sensitive to ischemia. On the other hand, BC is a reliable method of myocardial protection that has been worldwide and widely used in all areas of open-heart surgery for many years. Therefore, although various studies^[^1^-^10^]^ on DNC solutions used on adult patient populations have been published in the literature, there is no definite consensus about the superiority of either cardioplegia methods over each other or which one should be preferred.

Since 2018, the DNC solution has been used in all open-heart operations in our clinic. This study aimed to examine the impacts of BC and DNC solutions - which we used in isolated coronary artery bypass grafting (CABG) - on early mortality and major adverse events (MAE).

## METHODS

### Patient Population

Ethics committee approval was obtained from the institutional review board (2021/17-255). Since this is a retrospective study, informed consent was not obtained. Three hundred twenty-nine consecutive patients who underwent CABG in our clinic between January 2016 and January 2020 were retrospectively analyzed. Isolated CABG patients were included in the study, while patients who had valve replacement or repair together with CABG and off-pump CABG patients were excluded. Emergency operations, patients with an ejection fraction < 35%, patients over 85 years of age, patients with preoperative acute myocardial infarction (MI) or stroke, patients with acute or chronic renal failure, and reoperations were excluded as well. All patients were evaluated preoperatively using carotid doppler ultrasound. Patients who required carotid endarterectomy were excluded.

### Definitions

Complications were determined according to the Society of Thoracic Surgeons (or STS) criteria^[^11^]^. Mortality was defined as death during the time in hospital or within 30 days of discharge, stroke as a neurological deficit lasting > 72 hours, renal failure as creatinine values > 2.0 mg/dL or a doubling of the last measured creatinine, and prolonged mechanical ventilation (MV) as the duration of MV lasting > 24 hours. MI was determined as possible with ST-elevation or newly developing Q wave on electrocardiogram and creatine kinase-myoglobin binding value > 40 IU/L. European System for Cardiac Operative Risk Evaluation (EuroSCORE) was used for mortality risk scoring^[^12^]^.

Reoperation, cardiac tamponade, stroke, renal failure, need for extracorporeal membrane oxygenation (ECMO), and cardiopulmonary resuscitation (CPR) were defined as MAE.

### Cardiopulmonary Bypass Protocol

Medtronic Affinity (Medtronic Operational Headquarters, Minneapolis, Minnesota, United States of America) oxygenator and reservoir were used for the cardiopulmonary bypass (CPB) system. Sets were washed with prime solution. The prime solution consists of Ringer’s lactate solution and 20% mannitol solution. CPB flow was calculated based on patients’ body surface areas with a cardiac index of 2.4 L/min/m2. Ultrafiltration was used in all patients during CPB. All operations were performed under mild hypothermia (32-34°C). A heparin loading dose was administered with an activated clotting time of > 480 seconds. Heparin reversal was performed using protamine sulfate. During CPB, blood pH was aimed to be 7.35-7.40, PaO2 to be > 200 mmHg, and PaCo2 to be 5-40 mmHg.

### Surgical Technique

All patients were operated on under standard CPB after median sternotomy and aortic arterial and two-stage right atrial venous cannulation. In all patients, the left internal mammary artery was used as a left anterior descending artery bypass graft, and the great saphenous vein was used for the other coronary vessels. All distal anastomoses were performed under the aortic cross-clamp (ACC), and all proximal anastomoses were performed under side-clamping in the beating heart after removing ACC. Antegrade cardioplegia was used in all patients. Retrograde cardioplegia was not used in any patient. DNC solution was prepared at 4°C and BC at 24-28°C. DNC was administered as a single dose of 20 mL/kg (maximum 1000 mL) in all patients. BC was administered as the first dose of 15 mL/kg and repeated every 20 minutes at a dose of 5-10 mL/kg. Topical cooling was not administered in any group.

DNC was mixed with 26 mL of KCl2 mEq/L 7.5%, 13 mL of NaHCO3 8.4%, 13 mL of lidocaine 1%, 4 mL of MgSO4 50%, 16.3 mL of mannitol 20%, and 1000 mL of Plasma-Lyte A and ¼ of the patient’s blood, and the temperature was decreased 4°C. BC was prepared with an average of 200-300 cc of BC, and 1 mEq of potassium was added for every 100 cc of blood.

### Statistical Analysis

Analyses were performed using IBM Corp. Released 2011, IBM SPSS Statistics for Windows, version 20.0, Armonk, NY: IBM Corp., at a 95% confidence level. The suitability of the data belonging to the quantitative variables to the normal distribution was examined, and it was determined that they were suitable for normal distribution. Differences of quantitative variables according to groups were analyzed by independent groups *t*-test, and the relationship between qualitative variables and groups was analyzed by chi-square analysis. A *P*-value of < 0.05 was considered statistically significant.

## RESULTS

Three hundred twenty-nine isolated CABG were performed between the dates specified in our center. The group in which DNC was used was named Group D, and the group in which BC was used was named Group B. Group D consisted of 181 (55%) patients, and Group B consisted of 141 (45%) patients.

### Demographic and Preoperative Data

The mean age of the patients was 63.5±9.3 years, mean body weight was 78.0±11.4 kilograms, and mean body surface area was 1.85±0.16 m2. Two hundred forty-two (73.6%) of the patients were male, and 87 (26.4%) were female. No statistically significant difference was determined between the groups regarding age, weight, body surface area, and gender (*P*=0.615, *P*=0.560, *P*=0.934, *P*=0.365, respectively). The incidences of hypertension and chronic obstructive pulmonary disease were higher in Group D (*P*=0.001, *P*=0.001, respectively). Diabetes and smoking rates were higher in Group B (*P*=0.001, *P*=0.001, respectively). However, there was no statistical difference between the groups in terms of the EuroSCORE of the patients (*P*=0.955). The mean lactate level measured preoperatively was 2.44±2.50 mmol/L. Preoperative lactate levels were higher in Group B (*P*=0.001) (Table 1).

**Table 1:** Baseline characteristics of the patients.

Variables	All (n=329)	Group D (n=181)	Group B (n=148)	*P*-value
Age (years)	63.5±9.3	63.3±9.6	63.8±9.0	0.615
Weight (kg)	78.0±11.4	77.6±11.3	78.4±11.5	0.560
BSA (m2)	1.85±0.16	1.85±0.15	1.85±0.16	0.934
Gender				0.365
Male	242 (73.6%)	135 (74.6%)	107 (72.3%)	
Female	87 (26.4%)	46 (25.4%)	41 (27.7%)	
HT	260 (79%)	158 (87.3%)	102 (68.9%)	0.001
DM	106 (32.2%)	46 (25.4%)	60 (40.5%)	0.001
Smoking	173 (52.6%)	76 (42.0%)	97 (65.5%)	0.001
COPD	27 (8.2%)	16 (8.8%)	11 (7.4%)	0.001
EuroSCORE				0.955
1	102 (31%)	58 (32.0%)	44 (29.7%)	
2	86 (26.1%)	45 (24.9%)	41 (27.7%)	
3	22 (6.6%)	11 (6.1%)	11 (7.4%)	
4	42 (12.8%)	22 (12.2%)	20 (13.5%)	
5	37 (11.2%)	23 (12.7%)	14 (9.5%)	
6	34 (10.3%)	19 (10.5%)	15 (10.1%)	
7	6 (1.8%)	3 (1.7%)	3 (2.0%)	
Perioperative lactate (mmol/L)	2.44±2.50	1.98±2.68	3.00±2.13	0.001

BSA=body surface area; COPD=chronic obstructive pulmonary disease; DM=diabetes mellitus; EuroSCORE=European System for Cardiac Operative Risk Evaluation; HT=hypertension

### Operative Data

Mean CPB duration of the patients was 85.6±23.6 minutes. CPB duration was longer in Group B, being 80.2±20.8 minutes in Group D and 92.1±25.2 in Group B (*P*=0.001). Mean ACC duration was 44.8±13.1 minutes, and no statistically significant difference was found between the groups. Mean flow rate was 4433.7±361.9 mL, mean arterial pressure was 60.7±16.7 mmHg, mean intraoperative urine output was 296±311 mL, mean ultrafiltration volume during CPB was 1605.0±244.5 mL, and the lowest average temperature during the operation was 31.1±1.3°C (*P*=0.845, *P*=0.773, *P*=0.234, *P*=0.889, and *P*=0.374, respectively).

Mean lactate value after ACC was 2.8±2.8 mmol/L. After ACC, lactate was 2.5±2.6 mmol/L in Group D and 3.1±3.0 mmol/L in Group B (*P*=0.05). Mean lactate value measured after CPB was 2.85±2.58 mmol/L, and there was no statistically significant difference between the groups (*P*=0.553) (Table 2).

**Table 2:** Operative variables.

Variables	All (n=329)	Group D (n=181)	Group B (n=148)	*P*-value
CPB time (min)	85.6±23.6	80.2±20.8	92.1±25.2	0.001
ACC time (min)	44.8±13.1	45.1±13.1	44.5±13.2	0.712
Flow rate (mL/min)	4433.7±361.9	4437.2±373.3	4429.4±348.7	0.845
Mean arterial pressure (mmHg)	60.7±16.7	60.4±17.0	61.0±16.4	0.773
Urine output (ml)	296±311	278±304	319±318	0.234
Ultrafiltration volume (ml)	1605.0±244.5	1603.3±235.2	1607.1±256.3	0.889
Lowest temperature (°C)	31.1±1.3	31.1±1.3	31.2±1.3	0.374
Lactate level (mmol/L)				
After ACC	2.8±2.8	2.5±2.6	3.1±3.0	0.05
After separation of CPB	2.85±2.58	2.78±2.73	2.95±2.37	0.553

ACC=aortic cross-clamp; CPB=cardiopulmonary bypass

### Postoperative Complications

The most common complications were respiratory complications (*e.g.*, atelectasis, pleural effusion, pneumothorax). Respiratory complications were determined in 48 (14.5%) patients (*P*=0.530). Infective complications were observed in 44 (13.3%) patients, wound complications in 28 (8.5%) patients, arrhythmia in seven (2.1%) patients, and mediastinitis in six (1.8%) patients (*P*=0.494, *P*=0.396, *P*=0.120, and *P*=0.559, respectively). Six (3.3%) patients in Group D and four (2.7%) patients in Group B needed postoperative intra-aortic balloon pump (*P*=0.505).

MAE was observed in a total of 62 (18.8%) patients. MAE was determined in 30 (16.6%) patients in Group D and 32 (21.6%) patients in Group B (*P*=0.153). Postoperative ECMO support was provided to a total of 22 (6.6%) patients. Eleven (6.1%) patients in Group D and 11 (7.4%) patients in Group B had ECMO requirements (*P*=0.392). A total of nine (2.7%) patients had a postoperative MI. Five (2.8%) patients in Group D and four (2.7%) patients in Group B had MI (*P*=0.602). Angiographic findings included incorrect graft anastomosis, graft spasm, acute coronary artery thrombotic occlusion, and ischaemia due to incomplete revascularization. Emergency reoperation (redo) was performed in three (33.3%), acute percutaneous coronary intervention in four (44.4%), and conservative treatment (non-op.) in two (22.2%) patients. CPR was applied to five (2.8%) patients in Group D and seven (4.7%) patients in Group B (*P*=0.257). Renal failure developed in two (1.1%) patients in Group D and three (2.0%) patients in Group B (*P*=0.406). While the requirement for reoperation occurred in eight (4.4%) patients in Group D, it was observed in three (2.0%) patients in Group B (*P*=0.187). Stroke was determined in six (3.3%) patients in Group D and 13 (8.8%) patients in Group B. The intergroup difference was statistically significant (*P*=0.03). The total mortality was 12 (3.6%). Mortality was observed in nine (5.0%) patients in Group D and three (2.0%) patients in Group B (*P*=0.130).

While the lactate values measured at the postoperative 2^nd^ hour were 3.64±3.16 in Group B, they were 2.90±2.38 mmol/L in Group D (*P*=0.016). However, no statistically significant difference was found between the groups regarding lactate values measured at the postoperative 24^th^ hour (*P*=0.684).

Mean MV duration was 1.2±0.5 days, duration of intensive care unit (ICU) stay was 4.5±2.0 days, and hospital stay was 13.4±3.6 days. There was no statistically significant difference between the groups in terms of duration of MV, ICU stay, or hospital stay (*P*=0.689, *P*=0.710, *P*=0.613, respectively) (Table 3).

**Table 3:** Postoperative variables and complications.

Variables (%)	All (n=329)	Group D (n=181)	Group B (n=148)	*P*-value
MAE	62 (18.8%)	30 (16.6%)	32 (21.6%)	0.153
ECMO	22 (6.6%)	11 (6.1%)	11 (7.4%)	0.392
MI	9 (2.7%)	5 (2.8%)	4 (2.7%)	0.602
Stroke	19 (5.8%)	6 (3.3%)	13 (8.8%)	0.030
Renal failure	5 (1.5%)	2 (1.1%)	3 (2.0%)	0.406
Reoperation	11 (3.3%)	8 (4.4%)	3 (2.0%)	0.187
CPR	12 (3.6%)	5 (2.8%)	7 (4.7%)	0.257
Death	12 (3.6%)	9 (5.0%)	3 (2.0%)	0.130
Mediastinitis	6 (1.8%)	3 (1.7%)	3 (2.0%)	0.559
IABP	10 (3%)	6 (3.3%)	4 (2.7%)	0.505
Arrythmia	7 (2.1%)	3 (1.7%)	4 (2.7%)	0.120
Respiratory complication	48 (14.5%)	26 (14.3%)	22 (14.8%)	0.530
Infection complication	44 (13.3%)	25 (13.8%)	19 (12.8%)	0.494
Wound complication	28 (8.5%)	16 (8.8%)	12 (8.1%)	0.396
Lactate level				
Postoperative 2^nd^ hour	3.23±2.78	2.90±2.38	3.64±3.16	0.016
Postoperative 24^th^ hour	3.0±3.5	2.9±3.1	3.1±3.9	0.684
Ventilation time (days)	1.2±0.5	1.2±0.5	1.2±0.5	0.689
ICU time (days)	4.5±2.0	4.4±2.0	4.5±2.1	0.710
LOHS (days)	13.4±3.6	13.5±4.0	13.3±3.2	0.613

CPR=cardiopulmonary resuscitation; ECMO=extracorporeal membrane oxygenation; IABP=intra-aortic balloon pump; ICU=intensive care unit; LOHS=length of hospital stay; MAE=major adverse event; MI=myocardial infarction

## DISCUSSION

Our study aimed to compare the impacts of two different cardioplegia solutions on mortality and MAE in our clinic. Although stroke was more common in the BC group in patients undergoing isolated CABG, we did not observe a statistically significant difference between the groups in terms of total MAE or mortality. Moreover, we did not observe a statistically significant difference between the groups regarding the duration of MV, ICU stay, or hospital stay.

It was reported that the DNC solution has less inflammation and more antiapoptotic effect due to the lidocaine it contains^[^13^]^. Intracellular sodium and calcium accumulation decrease due to the low calcium content and magnesium sulfate content, as well as the blocking effect of lidocaine on sodium channels^[^14^,^15^]^. Concerns exist regarding the use of DNC solution in the adult patient population, as the pediatric patient group is more resistant to ischemia, and coronary artery disease is not included in this patient population. Again, there is a concern that the myocardium would warm up early and would not provide adequate protection due to the effect of washing it out by the coronary sinus blood if it is applied at long intervals. On the other hand, BC may provide better oxygen transfer and lower myocardial temperature, as it is repeated frequently, especially during prolonged ischemia. However, various studies have revealed that the DNC solution does not have a negative effect, and it has started to gain popularity in the adult patient group^[^1^-^3^,^16^,^17^]^.

Since the use of the DNC solution provides the surgeon with the opportunity to continue the operation uninterrupted and to be more focused during ACC, it is theoretically thought that it may be associated with shorter ACC time and CPB time. In most studies on this subject, it was reported that the duration of ACC and of CPB are shorter in the DNC group^[^1^-^3^,^9^,^10^]^. However, George et al.^[^4^]^ reported that they did not observe any differences between the groups regarding ACC and CPB durations in their study. Again, Guajardo et al.^[^18^]^, Ad et al.^[^6^]^, and Timek et al.^[^8^]^ reported that they found similar ACC duration in both groups. Similarly, we did not find a significant difference between the groups in terms of ABC in our study. However, the duration of CPB was shorter in the DNC group.

Studies on mortality and MAE have indicated similar results in the groups using both cardioplegia solutions^[^1^,^2^,^4^,^6^,^7^]^. Ad et al.^[^6^]^ and Yammine et al.^[^7^]^ reported similar results in both cardioplegia groups in their studies. Lenoir et al.^[^2^]^ found no difference between the groups in terms of mortality and postoperative complications, but they reported more myocardial damage in the DNC group, especially in patients with prolonged ischemic time. However, they did not observe its adverse clinical repercussions. The study by Misra et al.^[^1^]^ reported that they did not observe a significant difference between the groups in terms of mortality, postoperative left ventricular ejection fraction, acute renal failure, stroke, or low cardiac output syndrome. Again, George et al.^[^4^]^ reported that they did not observe any difference between the groups in terms of 30-day mortality or postoperative MI. We observed a higher stroke rate in the BC group in our study, but we did not determine any difference between the groups regarding total MAE or mortality.

The cardioplegia strategy may also affect the duration of MV, ICU stay, and hospital stay^[^1^,^4^]^. George et al.^[^4^]^ reported in a study in which they compared BC and DNC solutions used during valve operations that there was no difference between the groups in terms of duration of MV, duration of ICU stay, or length of hospital stay. Again, Misra et al.^[^1^]^, in their meta-analysis of 29 studies, reported no difference between the groups in which BC and DNC solutions were used in terms of duration of MV, ICU stay, or hospital stay. Similarly, our study did not observe a significant difference between the groups in terms of duration of MV, ICU stay, or hospital stay.

The incidence of postoperative arrhythmias in patients receiving DNC and BC is another matter of debate. In some previous studies no difference was found between DNC and BC groups^[^18^,^19^]^. However, Sanri et al.^[^20^]^ observed that the incidence of postoperative atrial fibrillation was lower in the DNC group in isolated CABG patients. However, we did not observe a statistically significant difference between the groups in terms of postoperative arrhythmia incidence in our study.

Apart from mortality, MAE, and ACC durations, DNC and BC solutions affect postoperative troponin values, left ventricular ejection fraction, and inotrope scores^[^1^-^4^]^. However, in our study, we focused on factors related to cardiac morbidity, such as MAE, mortality, duration of MV, and length of ICU and hospital stay, rather than direct laboratory findings of cardiac damage. Furthermore, in our study, we observed that lactate values measured after ACC and at the postoperative 2^nd^ hour were higher in the BC group. This finding might be related to the higher mean lactate values in the preoperative period in the BC group.

### Limitations

The main limitation of our study is that it is a single-center observational retrospective study. Furthermore, the fact that some preoperative and operative parameters (preoperative lactate, comorbidities, CPB duration) of the patients were not similar makes it difficult to conclude the development of postoperative complications. In addition, since patients with an ejection fraction of < 35% were excluded from the study due to the concern that it might have an impact on mortality and morbidity, we could not examine the effects of cardioplegia strategies used in this patient group.

## CONCLUSION

There was no significant difference in MAE, mortality, duration of MV, ICU stay, and hospital stay between the DNC and BC groups. We believe that both solutions can be used safely for cardiac protection in the adult patient population.
